# Dietary fiber supplementation mitigates gestational diabetes risk and preterm birth via gut microbiota modulation: a randomized controlled trial

**DOI:** 10.3389/fendo.2026.1794560

**Published:** 2026-03-30

**Authors:** Dongyao Zhang, Jing Sheng, Pengyuan He, Junjie Wang, Mengjie Zhou, Yixuan Sun, Yanan Cao, Ying Jiang, Haoyi Jia, Li Wang, Xianming Xu, Yincheng Teng

**Affiliations:** 1 Department of Gynecology and Obstetrics, Shanghai Sixth People’s Hospital Affiliated to Shanghai Jiao Tong University School of Medicine, Shanghai, China; 2 Department of Radiology, School of Medicine, Shanghai Pulmonary Hospital, Tongji University, Shanghai, China; 3 Department of Obstetrics and Gynecology, Shanghai General Hospital, Shanghai Jiao Tong University School of Medicine, Shanghai, China; 4 Nursing Department, Shanghai General Hospital, Shanghai Jiao Tong University School of Medicine, Shanghai, China

**Keywords:** dietary fiber, gestational diabetes mellitus, glucose metabolism, gut microbiota, weight gain

## Abstract

**Background:**

Gestational Diabetes Mellitus (GDM) poses severe health risks to mother and child, yet effective, non-invasive preventive strategies remain elusive. While the gut microbiota is known to influence glucose metabolism, its potential as a therapeutic target and predictive biomarker in high-risk pregnancies is underexplored. This study investigated whether soluble dietary fiber supplementation could remodel the gut microbiome to prevent GDM and improve pregnancy outcomes.

**Methods:**

We performed a single-center, randomized controlled trial with 98 pregnant women at elevated risk for GDM. For 5 weeks, from 20 to 24^+6^ weeks of pregnancy, participants were randomly assigned to either a fiber group (getting soluble fiber supplements every day) or a control group (getting normal care). Clinical outcomes encompassed OGTT results, gestational weight gain (GWG), and delivery outcomes. We used 16S rRNA sequencing to look at changes in gut flora. Furthermore, we developed a novel nomogram integrating clinical variables with microbial signatures to predict GDM risk.

**Results:**

Although GDM incidence did not statistically differ, the fiber group exhibited significantly improved glycemic excursions (predominantly lower 1h-PG, and reduced whole-OGTT glucose AUC and iAUC), reduced GWG during the 5-week intervention period (1.83 vs. 2.54 kg; *P* = 0.016), and a complete absence of preterm births (0% vs. 12.0%; *P* = 0.040). Microbiome analysis revealed that fiber intake enriched *Bifidobacterium* and *Limosilactobacillus* while suppressing *Phascolarctobacterium*. Functional prediction indicated a downregulation of inflammation-related pathways (HIF-1, AMPK) in the Fiber group. Crucially, a prediction model combining clinical factors with a specific “micro-balance” (*Bifidobacterium* ratio) achieved superior predictive accuracy (AUC 0.821) compared to clinical factors alone.

**Conclusions:**

Preliminary findings suggest that dietary fiber supplementation serves as a potent “biotic” intervention in high-risk pregnancies, improving 1-hour postprandial glucose homeostasis and eliminating preterm birth in this cohort. The mechanism appears associated with the specific enrichment of *Bifidobacterium*. Additionally, we validated a novel clinical-microbial nomogram, suggesting that integrating gut microbiome data can significantly enhance GDM risk stratification. Future extensive research is need to confirm these results.

## 1 Introduction

Gestational diabetes mellitus (GDM), defined as glucose intolerance initially identified during pregnancy, is a prevalent complication affecting 7–10% of pregnancies globally (1, 2). Its incidence is rising alongside economic development and lifestyle changes. GDM is associated with significant adverse outcomes, including pregnancy-induced hypertension, preterm delivery, macrosomia, and an elevated long-term risk of type 2 diabetes mellitus (T2DM) for both mother and offspring (3). Consequently, developing preventive strategies is critical, particularly for women with high-risk factors such as advanced maternal age, obesity, or a family history of diabetes (4).

Emerging evidence implicates the gut microbiota in host glucose homeostasis (5). Su et al. (6) demonstrated that GDM patients exhibit distinct microbial signatures—specifically higher abundances of *Corynebacteriales* and *Bacteroidetes*—which correlate with insulin resistance. Furthermore, the Firmicutes/Bacteroidetes (F/B) ratio and microbial diversity are established biomarkers for T2DM (7).

Dietary fiber is described as plant foods that resist hydrolysis by human digestive enzymes, principally cellulose, hemicellulose, and lignin (8). Encouraged consumption of dietary fiber (whole grains, vegetables, fruits, legumes, nuts, and seeds) has been associated with numerous health benefits and has been recommended for chronic disease prevention, notably achieving appropriate weight gain, and reducing the risk of developing insulin resistance and glucose intolerance (9). For example, Hull et al. (10) conducted a 12-week randomized trial with 20 pregnant women, revealing that adherence to a high-fiber diet (27–32 g/day) resulted in 4.1 kg less body weight gain and 2.8 kg less fat accrual during the intervention period compared to the control group (~17 g/day). Additionally, at one year postpartum, these women retained less weight (0.35 kg vs. 4.4 kg). According to a meta-analysis by McRae et al., there was a statistically significant decrease in the relative risk (RR) of T2DM (RR = 0.81-0.85) among those with the highest versus lowest dietary fiber intake (11).

Research suggested that the benefits of dietary fiber consumption may stem from the metabolic activity, composition, and production of fermentative end products in the gut microbiome. Because dietary fiber can be fermented into short-chain fatty acids (SCFAs, e.g., butyrate, acetate, and propionate), which are involved in insulin resistance and anti-inflammatory, or because of its ability to promote the enrichment of SCFA-producing bacteria (*Prevotella* and *Bifidobacterium*) *(*12). Currently, a high percentage (~70%) of pregnant women appear to be consuming insufficient dietary fiber (13). In China, the Chinese Dietary Reference Intakes (DRIs) 2013 recommends a minimum daily intake of 25 g of dietary fiber during pregnancy; however, the average total daily intake of dietary fiber among Chinese pregnant women (14.9 g) is considerably lower than the recommended daily allowance (14). Thus, increasing the amount of dietary fiber intake during pregnancy appears to be of great importance, especially for women at high risk of GDM.

Given the potential of fiber to modulate the gut microbiome, this study aimed to determine whether increasing dietary fiber intake in women at high risk of GDM could prevent the condition’s onset and positively regulate the gut microbiota.

## 2 Methods

### 2.1 Study design and population

This single-center, randomized controlled trial (RCT) investigated the impact of dietary fiber on GDM prevention and gut microbiota alterations. The protocol was registered (ChiCTR2000036575) and approved by the Ethics Committee of Shanghai General Hospital (2020KY098), and adhered to the guidelines outlined in the Helsinki Declaration of 1975, as modified in 2000. Recruitment occurred between June 2021 and September 2022. Inclusion criteria were: (1) age: 18–50 years old; (2) singleton and natural pregnancy; (3) at least two of the following criteria are met: ① age ≥ 35 years old (advanced maternal age was included as it is a well-established independent risk factor for GDM, associated with progressive decline in pancreatic *β*-cell function and increased insulin resistance) (15); ② pre-pregnancy BMI ≥25 kg/m^2^; ③ family history of diabetes; ④ history of GDM or PCOS; ⑤ history of macrosomia delivery; (4) not taking antibiotics, probiotics, and other drugs or foods that may interfere with intestinal flora during pregnancy. Exclusion criteria: (1) history of hyperthyroidism, liver damage, pancreatic disease, and chronic cardiovascular and cerebrovascular diseases (2) pre-pregnancy diabetes; (3) taking glucocorticoids and other drugs that affect blood sugar levels during pregnancy; (4) being intolerant of dietary fiber supplements or losing follow-up during the study period. A sample size of 109 was calculated using standard statistical methods for comparing two independent proportions to detect a clinically significant reduction in GDM incidence (from 35% to 12%). These baseline assumptions for the power analysis (a power of 80%, a significance level of 5%, an allocation ratio of 1:1, and an attrition rate of 5%) were informed by previous dietary intervention datasets in pregnant cohorts (16). All participants willingly provided written informed consent to participate in this study.

### 2.2 Study conduct

**Figure 1:**
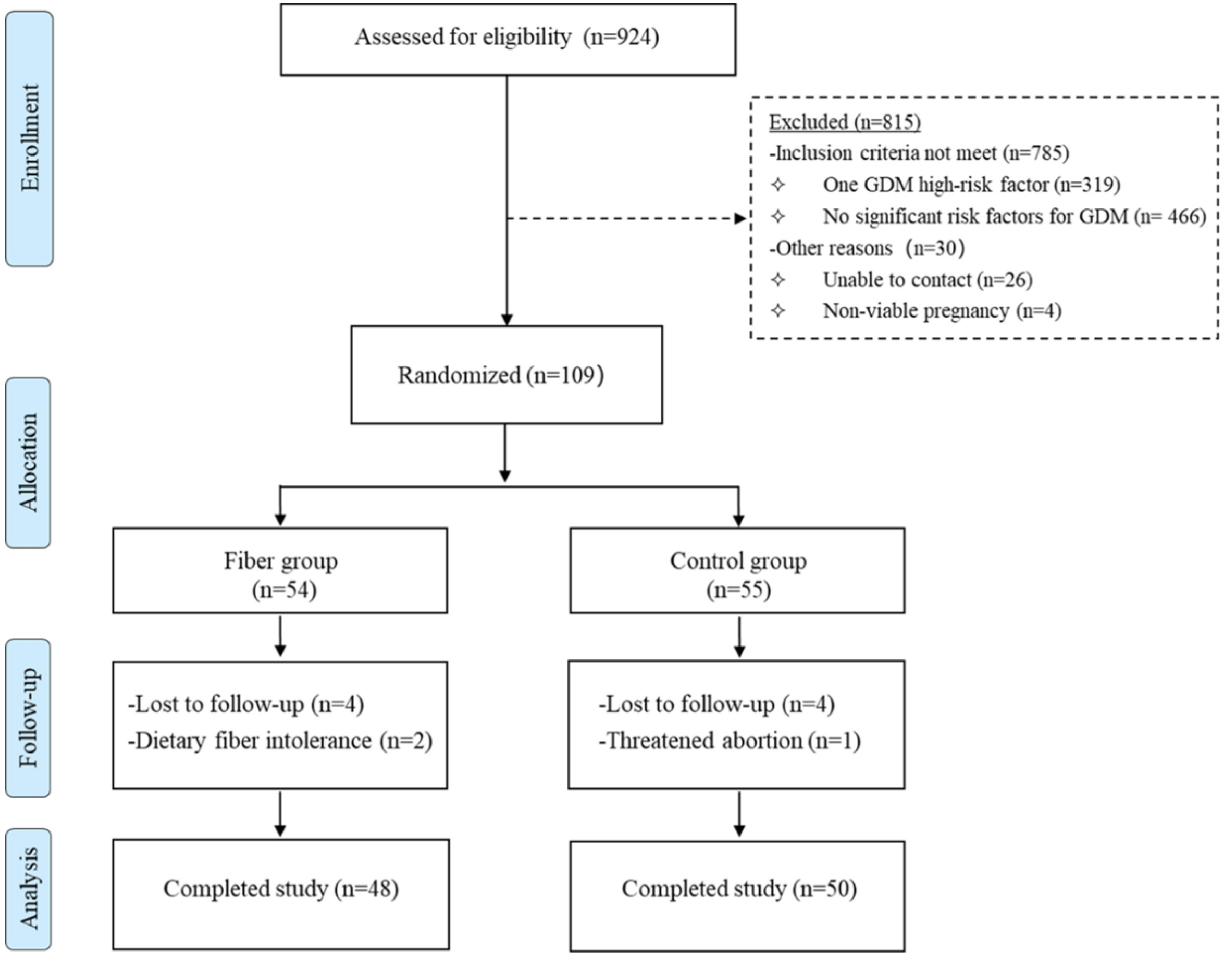
Flow chart of the screening process.

During the sampling process, we screened 924 people and, selected 109 candidates who met the criteria for the study (**Figure 1**). All women included in the study were randomized to the control group (n = 55) and the fiber group (n = 54) using stratified randomization by age (<35 or ≥35years old), GDM history (yes or no) and BMI (<30.0 or ≥30.0 kg/m^2^) after screening. Random coding table was undertaken using Excel random number generator (Microsoft Excel 2021). Women in the control group only received routine nursing care during pregnancy. In contrast, participants in the fiber group received two bags of soluble dietary fiber powder (Nutrasumma, Qingdao Nutrasumma Health Technology Co., Ltd.) daily from 20 to 24^+6^ weeks of gestation (refers to the period from 20 weeks and 0 days to 24 weeks and 6 days of gestation, representing a standardized 5-week intervention window). Participants were instructed to dissolve the contents of each sachet in 150–200 ml of warm water (to prevent clumping without altering the fiber composition), stirring the mixture until the powder was completely dissolved. Women in the fiber group were asked to record possible adverse effects related to supplements consumption daily, and the quantity of dietary fiber powder that was returned was used to determine adherence. Additionally, nutritionists offered nutrition education and dietary advice to both cohorts according to the Chinese Dietary Guidelines for Pregnant Women (17). The following recommendations are provided: (1) adopt a diet rich in iodized salt and iron-containing foods (20–50 g red meat per day); (2) increase milk intake to 500 g per day; (3) consume fish, poultry, eggs, and lean meat at a rate of 50 g per day; (4) maintain a healthy weight and engage in moderate physical activity for at least 30 minutes per day; and (5) quit smoking and maintain a positive attitude. All pregnant women enrolled in the study underwent a 75-g oral glucose tolerance test (OGTT) at 25–28 weeks of gestation, identifying GDM as satisfying any subsequent plasma glucose criteria: fasting plasma glucose (FPG)≥5.1 mmol/L, 1-hour plasma glucose (1h-PG)≥10.0 mmol/L, and 2-hour plasma glucose (2h-PG) ≥8.5 mmol/L (18).

### 2.3 Clinical data and biochemistry

Pregnant women’s ID cards provide maternal age (years). Self-reported height and weight determine pre-pregnancy BMI (kg/m^2^). Measurements of weight and resting blood pressure were taken at enrollment (<20 weeks), 20 weeks, and 25 weeks. We extracted maternal and neonatal data abstracted from the electronic medical record. Additional pieces of information were collected, including maternal pregnancy history, previous PCOS and GDM, first-degree relatives with diabetes, macrosomia delivery history, and so on.

We drew and evaluated antecubital vein blood at enrollment and 25–28 gestational weeks within 3 hours. FPG, 1hPG, and 2hPG were measured with a biochemical autoanalyzer (ADVIA2400 Chemistry System, Siemens Healthcare Diagnostics Ltd, Germany). Serum insulin was detected with a chemiluminescent immunoassay (Beckman Coulter Inc., Brea, CA, USA). An analyzer (HLC-723G8, Tosoh Corporation, Japan) calculated HbA1c using high-pressure liquid chromatography.

### 2.4 Sample collection, DNA extraction, and 16S rRNA sequencing

Before and after the intervention, researchers verbally explained the study to participants and provided comprehensive printed instructions for collecting stools. A swab and a fecal storage kit, which included 4 mL of Stool Preservation Solution from Bohao Biotechnology Co., Ltd. in Shanghai, China, were used for the microbiome sample. Thereafter, the samples were stored at −80 °C until DNA extraction. Qiagen QIAamp DNA Stool Mini Kits (Qiagen, California, USA) extracted total bacterial genomic DNA from all specimens. NanoDrop 2000 (Thermo Scientific, USA) measured the extracted DNA concentration. We excluded samples that did not meet detection requirements. The extracted DNA was amplified via polymerase chain reaction (PCR) using the following primers: 806R (5’-GGA CTA CHV GGG TWT CTA AT-3’) and 338F (5’-ACT CCT ACG GGA GGC AGC AG-3’) of the V3-V4 region of the bacterial 16S rRNA gene. The PCR products were extracted on a 2% agarose gel, purified with an AxyGen Biosciences centrifuge (Axygen Biosciences, Union City, CA, USA), and quantified with a Qubit4.0 thermofisher (USA). We used the refined amplified fragments to make a PE 2x300 library, following the Illumina MiSeq platform’s standard operating procedure (Illumina, San Diego, CA, USA). After that, the Illumina MiSeq PE300 platform sequenced the library.

### 2.5 Sequence analysis

We used Fastp (V0.20.0) for raw sequence quality control and Flash (V1.2.11) for splicing. More details are as follows:

1. Setting a 50-bp window. All sequences at the base’s back end were clipped from the window’s front end if the average quality value was< 20, and any sequences that remained > 50 bp after quality control were eliminated.
2. The base overlap spliced the two ends’ sequences, setting the maximum mismatch rate between overlaps at 0.2 for sequences >10 bp. The last few sequences were dropped.
3. A sequence was associated with a sample based on the barcode and primers at both ends of the sequence. During this process, we permitted two base mismatches in the primer, but the barcode had to match exactly.

The UPARSE tool (http://drive5.com/uparse/, version7.1) was performed to cluster OTU sequences based on a similarity threshold of 97%, and eliminate chimeras. Utilizing the RDP classifier (https://sourceforge.net/projects/rdp-classifier/, version 2.2) to annotate the species category for each sequence, the comparison threshold was set to 70% compared to the Silva database (SSU138).

### 2.6 Bioinformatics analysis

For microbiome diversity and composition analysis, we used the R packages ‘microeco’ and ‘phyloseq’. Analysis of α-diversity, including Chao1 index, and analysis of β-diversity, including principal coordinate analysis (PCoA) ranking analysis of Bray Curtis distance matrix, are performed with the R package for ‘vegan’. The linear discriminant analysis (LDA) effect size (LEfSE) (logarithmic LDA scores >2.0) was used to identify the key bacterial taxa that differed between the two groups. The correlation heatmap analysis was used to calculate the spearman correlation coefficient between environmental factors and the key differential species in the two groups, and a heatmap diagram was used to visually display the obtained numerical matrix. For 16S function prediction, we utilized PICRUSt to standardize the OTU abundance table. Subsequently, we obtained the corresponding Kyoto Encyclopedia of Genes and Genomes (KEGG) information for each OTU by referencing the KEGG database through Greengene ID. Finally, the R packages ‘ggplot’ and ‘pheatmap’ were used for the drawing of both box plots and heat maps.

### 2.7 Establishment and validation of the nomogram model

On the basis of baseline clinical variables gathered at enrollment (gestational age, blood pressure, gravidity/parity, pre-pregnancy overweight/obesity, family history of diabetes, previous GDM/PCOS, macrosomia history, age, BMI, FPG, HbA1c), we predetermined a concise clinical predictor set (Clin4): age, BMI, previous GDM (PGDM), and BPG. This decision was influenced by clinical accessibility during early pregnancy, previous evidence of a robust correlation with dysglycemia, and the control of overfitting due to 22 outcome events (events-per-variable around 5.5).

Microbiome processing and feature derivation. Post-intervention LEfSe and cladogram analyses were used to identify fiber-responsive lineages: To reduce redundancy, taxonomic features were collapsed to the genus level; when a genus was unavailable, the closest family was used as a proxy. Taxa with prevalence<15% or mean relative abundance<0.1% were excluded. To avoid information leakage, all microbiome features were derived from baseline samples only, where the above taxa were ranked by baseline relative abundance. The three highest-abundance candidates—*Bifidobacterium*, *Phascolarctobacterium*, and *Lachnoclostridium* were then combined into a single log-ratio balance feature: micro_balance = log(Bifidobacterium + ε) − ½ [log(Phascolarctobacterium + ε) + log(Lachnoclostridium + ε)]

with $\epsilon =10^{-6}$ added as a pseudocount. The significance of the ‘fiber-responsive’ niche increases with micro_balance values, which served as the microbiome predictor in subsequent models.

We fit three multivariable logistic regression models: (i) Clin-only (Clin4), (ii) Micro-only (micro-balance), and (iii) Combined (Clin4 + micro-balance). Analyses were performed in R (version 4.5.0) using rms (lrm/nomogram/calibrate/validate), pROC (ROC/AUC with DeLong CI), and rmda (decision curves). Model discrimination was quantified by the area under the ROC curve (AUC) with 95% confidence intervals (DeLong). Internal validation used bootstrap resampling (1000 draws) on the entire dataset to estimate optimism-corrected C-index (AUC), calibration slope, and intercept. This resampling method was chosen over a traditional split-sample approach to maximize the utility of our limited sample size and provide a more robust assessment of model performance. Calibration curves (apparent and bias-corrected vs. ideal) were generated from the bootstrap procedure. The combined model was visualized as a nomogram and a coefficient table/formula was provided for reproducibility. Decision curve analysis compared Clin-only, Micro-only, and Combined models across probability thresholds 0.00–0.60 (primary focus 0.10–0.30), reporting standardized net benefit. For case–control sampling, analyses were repeated specifying the target population prevalence when applicable.

### 2.8 Statistical analysis

Continuous data are presented as mean ± standard deviation (SD), and comparisons between two groups were made using the t-test or Mann–Whitney U test when appropriate. The categorical variable was expressed as n (%), and the χ^2^ test or Fisher’s exact test was applied, depending on the situation. Statistical analysis was performed by using Statistics, Version 27.0 (SPSS, Chicago, IL, USA) and R software (version 4.5.0, http://www.R-project.org/). The threshold for statistical significance was assumed to be *P* < 0.05.

## 3 Results

### 3.1 Baseline characteristics

In this study, ninety-eight participants completed it (control: n = 50; fiber: n = 48) (**Figure 1**). The pre-pregnancy BMIs of the fiber group and the control group were 25.29 and 25.65 kg/m², respectively, and their mean ages were 33.5 and 32.48 years, respectively. Pregnant women’s age and BMI before pregnancy did not differ substantially between the two groups (all P>0.05). Furthermore, no statistically significant variations were observed between the groups regarding the history of pregnancy and delivery, diabetes in the family, PCOS history, GDM history, and metabolic parameters such as blood sugar and blood lipids (all P>0.05). As shown in **Table 1**.

**Table 1:** Characteristics of the participants before the intervention.

Variables	Control group (n =50)	Fiber group (n=48)	*P* value
Gestational age at enrollment, weeks (mean ± SD)	12.81 ± 1.37	12.57 ± 1.51	0.405
Gestational age at OGTT, weeks (mean ± SD)	25.52 ± 1.25	25.67 ± 0.92	0.512
Age, years (mean ± SD)	33.5 ± 4.45	32.48 ± 4.49	0.261
BMI, kg/m^2^ (mean ± SD)	25.29 ± 4.31	25.65 ± 2.67	0.616
Systolic blood pressure, mmHg (mean ± SD)	118.82 ± 10.68	117.17 ± 11.35	0.459
Diastolic blood pressure, mmHg (mean ± SD)	71.76 ± 9.44	70.71 ± 8.93	0.573
Gravidity (mean ± SD)	2.80 ± 1.59	2.54 ± 1.29	0.371
Parity (mean ± SD)	0.90 ± 0.76	0.65 ± 0.67	0.083
Pre-pregnancy overweight or obesity, n (%)	36 (72.0)	38 (79.2)	0.410
Family history of diabetes, n (%)	14 (28.0)	21(43.8)	0.104
Previous GDM, n (%)	7 (14.0)	5 (10.4)	0.598
Previous PCOS, n (%)	15 (30.0)	13 (48.0)	0.749
Macrosomia delivery history, n (%)	13 (26.0)	6 (12.5)	0.091
HbA1c (mean ± SD)	5.29 ± 0.46	5.20 ± 0.25	0.204
FPG, mmol/L (mean ± SD)	4.56 ± 0.49	4.45 ± 0.37	0.188
TC, mmol/L (mean ± SD)	4.96 ± 0.79	4.80 ± 1.00	0.375
TG, mmol/L (mean ± SD)	1.70 ± 0.55	1.64 ± 0.55	0.598
HDL-C, mmol/L (mean ± SD)	1.58 ± 0.31	1.47 ± 0.37	0.132
LDL-C, mmol/L (mean ± SD)	2.65 ± 0.71	2.59 ± 0.74	0.703

Data are presented as mean ± SD or n (%). OGTT, oral glucose tolerance test; GDM, gestational diabetes mellitus; BMI, body mass index; PCOS, polycystic ovary syndrome; FPG, fasting plasma glucose; TC, total cholesterol; TG, triglyceride; HDL-C, high-density lipoprotein cholesterol; LDL-C, low-density lipoprotein cholesterol.

### 3.2 Clinical characteristics after intervention

An OGTT was conducted during the 25–28 weeks of the gestation period. Among the 50 women in the control group, 13 (26.0%) developed GDM, while 10 (20.8%) were in the fiber group. There was not a significant difference in the incidence of GDM between the two groups (P = 0.546). In terms of blood sugar metabolism, the levels of 1hPG were lower in women in the fiber group versus the control group (7.74 ± 2.13 vs. 7.81 ± 1.19; P = 0.015). Likewise, the values of 1hPG-FPG (4.38 ± 1.60 vs. 3.64 ± 1.13; P = 0.010), AUC (14.78 ± 2.61 vs. 13.68 ± 1.83; P = 0.018), and iAUC (5.79 ± 2.03 vs. 4.79 ± 1.58; P = 0.008) were lower in the intervention group compared with the control group. There were no significant differences in the levels of HbA1c, FPG, 2hPG, and other related indicators (all P>0.05). As presented in **Table 2**.

**Table 2:** Comparing OGTT results after intervention between groups.

Variables	Control group (n =50)	Fiber group (n=48)	*P* value
GDM, n (%)	13 (26.0)	10 (20.8)	0.546
HbA1c, (mean ± SD)	5.11 ± 0.43	5.00 ± 0.29	0.157
FPG, mmol/L (mean ± SD)	4.49 ± 0.61	4.44 ± 0.61	0.599
1hPG, mmol/L (mean ± SD)	8.88 ± 1.84	8.09 ± 1.26	0.015^*^
2hPG, mmol/L (mean ± SD)	7.32 ± 1.62	6.74 ± 1.62	0.082
FPG_25-20_ (mmol/L)	-0.07 ± 0.55	-0.01 ± 0.40	0.501
1hPG-FPG, mmol/L (mean ± SD)	4.38 ± 1.60	3.64 ± 1.13	0.010^*^
1hPG-2hPG, mmol/L (mean ± SD)	1.56 ± 1.53	1.34 ± 1.59	0.499
2hPG-FPG, mmol/L (mean ± SD)	2.83 ± 1.40	2.30 ± 1.56	0.084
AUCs (mean ± SD)	14.78 ± 2.61	13.68 ± 1.83	0.018^*^
iAUCs (mean ± SD)	5.79 ± 2.03	4.79 ± 1.58	0.008*
TC, mmol/L (mean ± SD)	5.87 ± 0.96	5.73 ± 1.12	0.496
TG, mmol/L (mean ± SD)	2.55 ± 0.75	2.51 ± 0.78	0.790
HDL-C, mmol/L (mean ± SD)	1.79 ± 0.39	1.75 ± 0.41	0.627
LDL-C, mmol/L (mean ± SD)	3.04 ± 0.84	3.00 ± 0.91	0.807
HOMA-IR (mean ± SD)	2.27 ± 1.23	2.71 ± 1.30	0.087

Data are presented as mean ± SD or n (%). *Significantly different values (*P* < 0.05). GDM, gestational diabetes mellitus; FPG, fasting plasma glucose; 1hPG, 1-hour blood glucose; 2hPG, 2-hour blood glucose; FPG_25-20_, fasting plasma glucose change between 20 and 25 weeks; 1hPG-FPG, the difference between 1-hour and fasting plasma glucose levels; 1hPG-2hPG, the difference between 1-hour and 2-hour plasma glucose levels; 2hPG-FPG, the difference between 2-hour and fasting plasma glucose levels; AUC, whole-OGTT glucose area under the curve; iAUC, whole-OGTT glucose incremental area under the curve; TC, total cholesterol; TG, triglyceride; HDL-C, high-density lipoprotein cholesterol; LDL-C, low-density lipoprotein cholesterol; HOMA-IR, homeostatic model assessment for insulin resistance.

### 3.3 Weight change

Based on the data provided in **Table 3**, there was no significant difference in the weight and BMI of women in the control group compared to those in the fiber group before the intervention (all *P*>0.05). Additionally, following a 5-week intervention with dietary fiber, no statistically significant disparity in weight or BMI was observed between the two groups (all *P*>0.05). However, the control group reported a significantly greater increase in weight gain and BMI during the intervention compared to the fiber group (2.54 ± 1.61 vs. 1.83 ± 1.21 and 1.01 ± 0.64 vs. 0.71 ± 0.48, respectively; all *P* < 0.005).

**Table 3:** Weight and BMI comparison between groups following the intervention.

Variables	Control group (n =50)	Fiber group (n=48)	*P* value
W_20_ (kg)	67.90 ± 12.07	70.43 ± 8.18	0.227
W_25_ (kg)	70.45 ± 12.13	72.26 ± 8.15	0.386
W_25-20_ (kg)	2.54 ± 1.61	1.83 ± 1.21	0.016^*^
BMI_20_ (kg/m^2^)	26.78 ± 4.08	27.25 ± 2.55	0.499
BMI_25_ (kg/m^2^)	27.79 ± 4.10	27.95 ± 2.55	0.808
BMI_25-20_ (kg/m^2^)	1.01 ± 0.64	0.71 ± 0.48	0.011^*^

Data are presented as mean ± SD. *Significantly different values (*P* < 0.05). W_20_, maternal weight at 20 weeks; W_25_, maternal weight at 25 weeks; W_25-20,_ maternal weight gain between 20 and 25weeks; BMI_20_, body mass index at 20 weeks; BMI_25_, body mass index at 25 weeks; BMI_25-20_, body mass index gain between 20 and 25weeks.

### 3.4 Maternal pregnancy and birth outcomes

Compared to the control group, none of the participants in the fiber group suffered premature delivery. In contrast, the control group had a preterm birth rate of 12.0% (6 out of 50), and this difference was statistically significant (P = 0.040). The mean gestational weeks in the fiber group were 39.04, which was considerably higher than the control group’s average of 38.33 weeks (P = 0.004). In addition, no significant differences were observed in the other maternal pregnancy or birth outcomes between the intervention and the control group (all P>0.05). As shown in **Table 4** (19).

**Table 4:** Pregnancy outcomes in both groups.

Variables	Control group (n =50)	Fiber group (n=48)	*P* value
Maternal			
Gestational hypertension, n (%)	3 (6.0)	1 (2.1)	0.639
Pre-eclampsia, n (%)	1 (2.0)	1 (2.1)	1.000
Polyhydramnios, n (%)	1 (2.0)	0 (0)	1.000
Premature rupture of membranes, n (%)	8 (16.0)	8 (16.7)	0.929
Postpartum hemorrhage, n (%)	3 (6.0)	1 (2.1)	0.639
Excessive weight gain※, n (%)	17 (34.0)	25 (52.1)	0.071
Inadequate weight gain※, n (%)	9 (18.0)	8 (16.7)	0.862
Weekly weight gain after enrollment (kg)	0.42 ± 0.15	0.43 ± 0.17	0.675
Cesarean section, n (%)	30 (60.0)	26 (54.2)	0.560
Neonatal			
Gestational age at delivery (weeks)	38.33 ± 1.47	39.04 ± 0.90	0.004
Preterm (<37 weeks), n (%)	6 (12.2)	0 (0.0)	0.040
Birth weight (g)	3284.00 ± 395.04	3416.46 ± 416.95	0.110
Macrosomia (≥4000 g)	1 (2.0)	3 (6.3)	0.581
Small for gestational age (<2500 g)	1 (2.0)	2 (4.2)	0.971

Data are presented as mean ± SD or n (%). *Significantly different values (*P* < 0.05). ※Excessive weight gain and inadequate weight gain are defined as gestational weight gain that falls above or below recommended ranges. According to IOM recommendations: gestational weight gain for underweight (BMI<18.5 kg/m^2^), normal weight (18.5 kg/m^2^≤ BMI< 24.9 kg/m^2^), overweight (25 kg/m^2^≤ BMI< 29.9 kg/m^2^), and obese women (BMI≥30 kg/m^2^) were 12.5–18 kg, 11.5–16 kg, 7-11.5 kg, and 5–9 kg, respectively.

### 3.5 Gut microbiota composition

**Figure 2:**
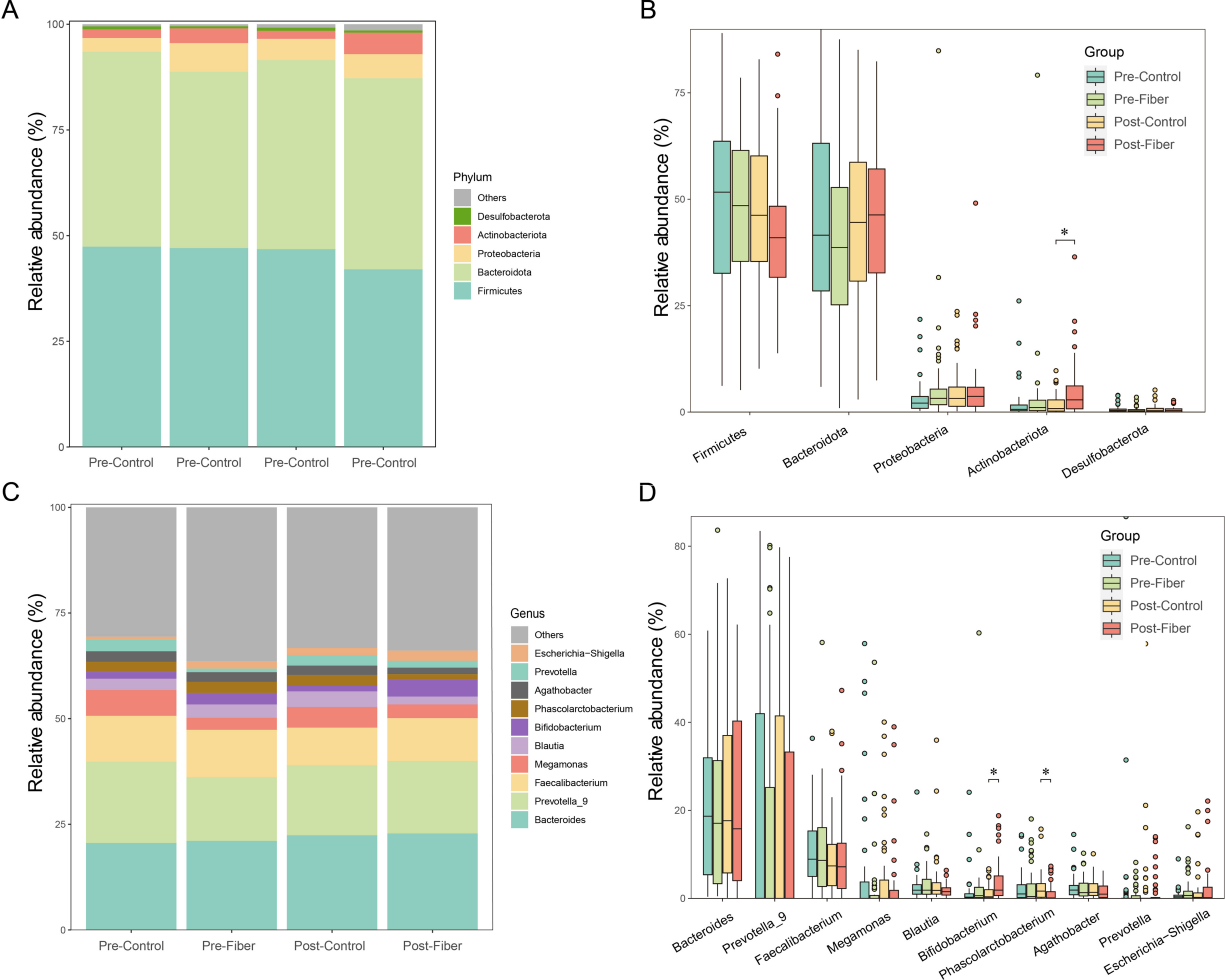
Effects of fiber on gut microbiota composition and relative abundance at various taxonomic levels in the two groups. **(A, B)** Composition and relative abundance of dominant phyla between the two groups pre- and post-intervention. **(C, D)** Composition and relative abundance of dominant genus between the two groups pre- and post-intervention *P <0.05.

Then, we analyzed the changes of gut microbiome at different taxonomic levels. At the phylum level, Firmicutes, Bacteroidetes, Proteobacteria, Actinobacteria, and Desulfobacterota represented the dominant taxa across all samples (**Figure 2A**). (Note: Under the recently updated and validated bacterial taxonomy, *Firmicutes*, *Bacteroidetes*, *Proteobacteria*, and *Actinobacteria* are now formally designated as *Bacillota*, *Bacteroidota*, *Pseudomonadota*, and *Actinomycetota*, respectively) (20). Among these taxa, *Firmicutes* were the most prevalent phyla in the two groups, constituting a mean relative abundance of 47.2%. On the contrary, the fiber group exhibited the highest prevalence of *Bacteroidetes* at 45.2% relative abundance after intervention, and *Firmicutes* followed with 42.0%, while the control group failed to show any noticeable change. Further analysis revealed that the fiber group showed significant increases in relative abundance of *Actinobacteriota* compared with the control group post-intervention *(P* < 0.05), and there are no significant differences in other relative abundances at the phylum level before and after intervention (all *P*>0.05) (**Figure 2B**).

At the genus level, we found that the three most common taxa in both groups were *Bacteroides, Prevotella_9, and Faecalibacterium* before and following the intervention (**Figure 2C**). Additionally, the present study indicated that the relative abundance of *Bifidobacterium* in the fiber group was significantly higher than that in the control group post intervention, while the relative abundances of *Phascolarctobacterium* were reduced (all *P* < 0.05). No significant differences were found in the genus level at baseline (all *P*>0.05). The top ten genera in the two groups are displayed in **Figure 2D**.

### 3.6 Gut microbiota differences

**Figure 3:**
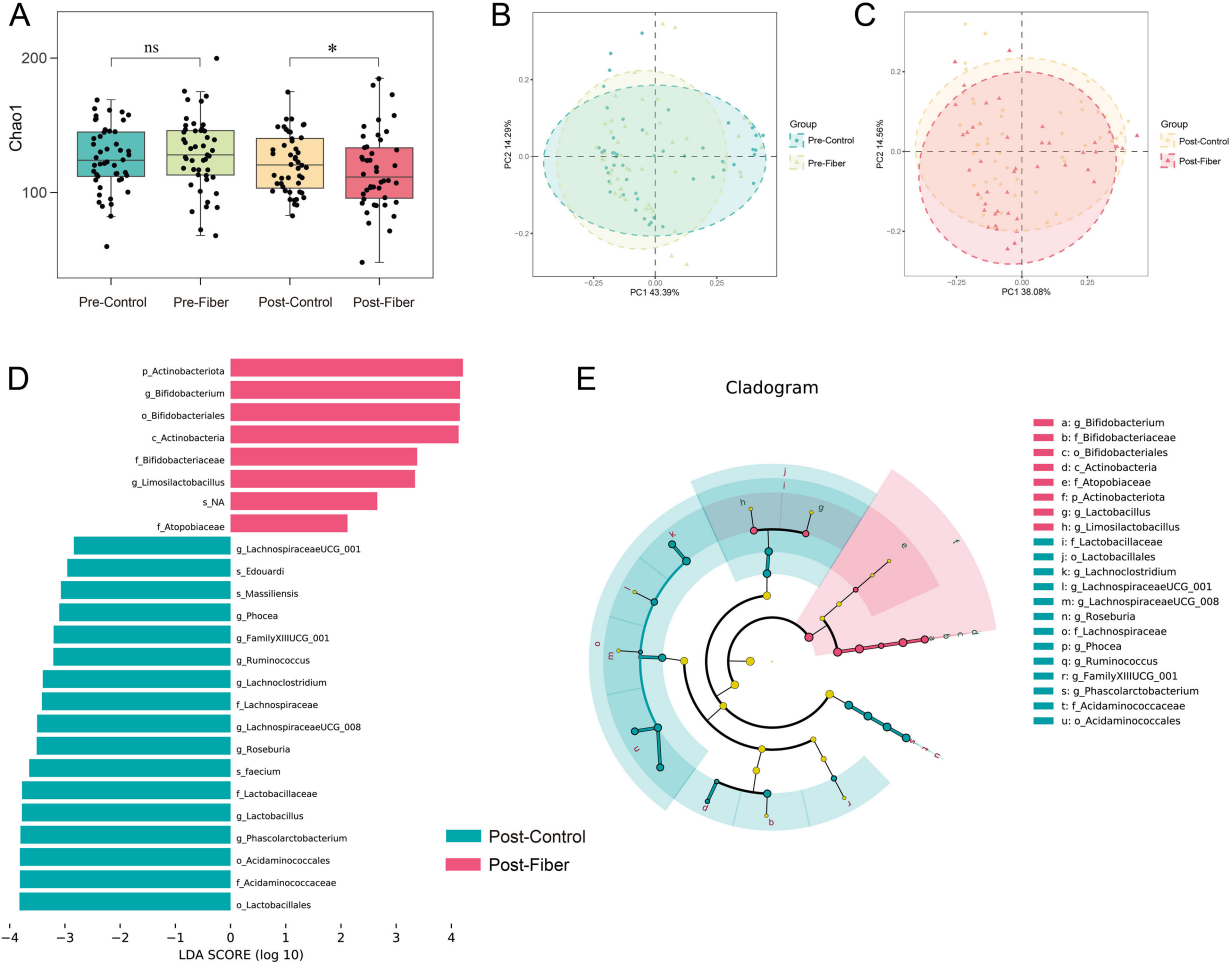
Diversity analysis of gut microbiota among between groups. **(A)** Chao1 indexes in control group are significantly higher than that in fiber group (*P* < 0.05). **(B, C)** PCoA analysis indicated there was no significant difference between the two groups pre- and post-intervention. **(D)** LDA score (logarithmic LDA scores >2.0) of microbial features that are differential between the fiber and the control groups post intervention. **(E)** Cladogram showing a taxonomic representation of the differences between the two groups post intervention. Red indicates higher score in fiber group and green indicates higher score in control group. PCoA, principal coordinate analysis; LDA, the linear discriminant analysis *P <0.05.

To further explore the altered gut microbiota between groups, the study exhibited a significant reduction in α-diversity (Chao1 index: *P* = 0.011) in the fiber group when compared with those in the control post-intervention (**Figure 3A**). At the same time, the β-diversity was not significantly different between groups (*P*>0.05) (**Figures 3B, C**). Next, at the threshold of 2, we used LEfSe to identify the key differential taxa between the two groups after intervention. A total of 1 phylum, 1 class, 3 orders, 5 families, 11 genera, and 4 species were found to be significantly different between the two groups (**Figure 3D**). At the genus level, we found that the abundances of *LachnospiraceaeUCG-001*, *Phocea*, *FamilyXllUCG-001*, *Ruminococcus*, *Lachnoclostridium*, *LachnospiraceaeUCG-008*, *Roseburia*, *Lactobacillus*, and *Phascolarctobacterium* were significantly lower, and the abundances of *Limosilactobacillus*, and *Bifidobacterium* were significantly higher in women who received dietary fiber powder than those in the control group. The cladogram depicts a taxonomic representation of the variations between fiber and the control group (**Figure 3E**).

### 3.7 Correlation between gut microbiota and clinical characteristics

**Figure 4:**
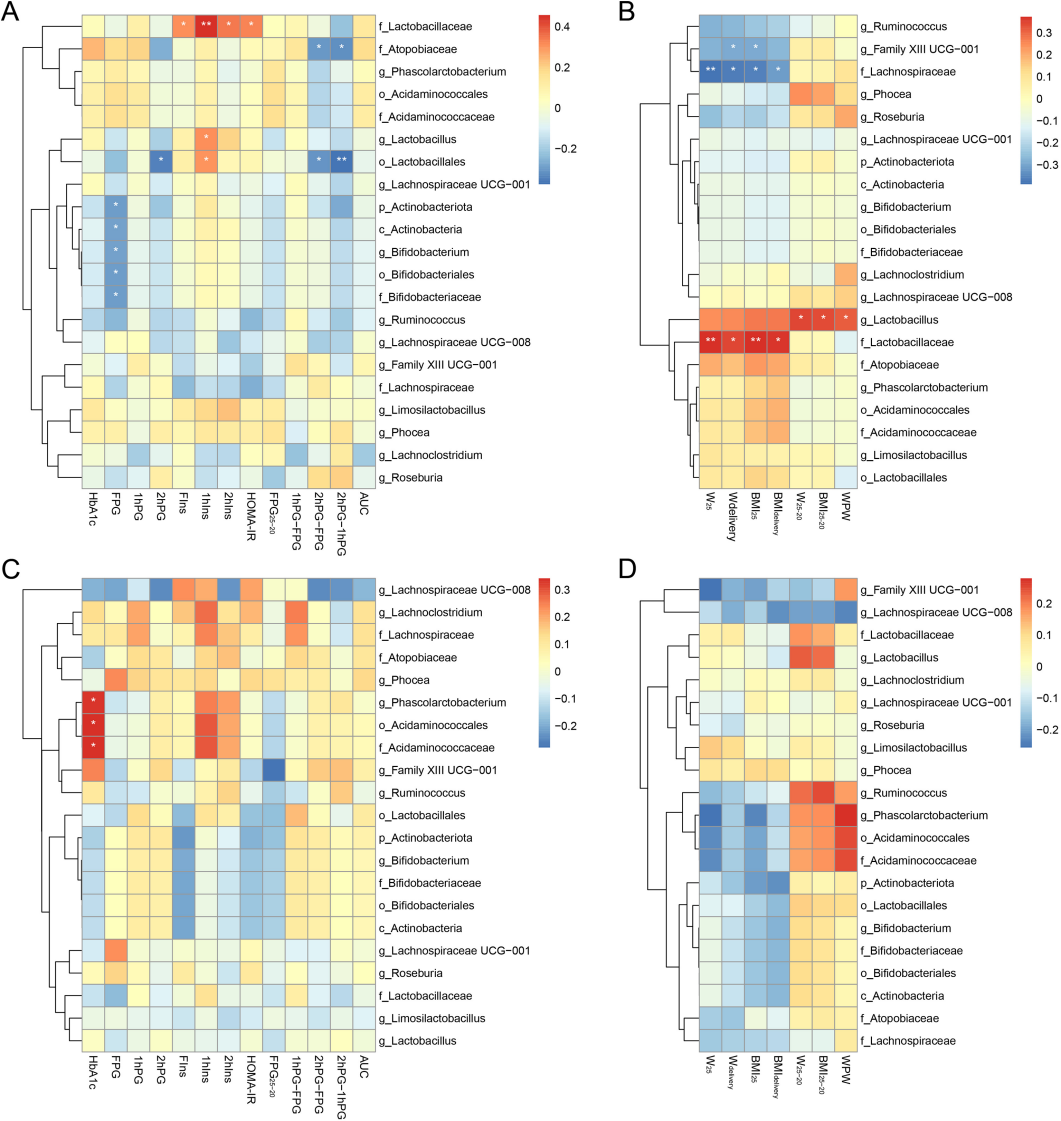
The Spearman correlation heat map between the clinical characteristics and the dominant taxon that differed between the two groups post intervention. The Spearman Correlation Heatmap between glucose metabolism levels (HbA1c, FPG, 1hPG, 2hPG, FIns, 1hIns, 2hIns, HOMA-IR, FPG_25-20_, 1hPG-FP, 2hPG-FPG, 2hPG-1hPG, and AUC) and key differential gut microbiota in control **(A)** and fiber group **(C)**. The Spearman Correlation Heatmap between weight levels (W_25_, W_delivery_, BMI_25_, BMI_delivery_, W_25-20_, BMI_25-20_, and WPW) and key differential gut microbiota in control **(B)** and fiber group **(D)**. FPG, fasting plasma glucose; 1hPG, 1-hour blood glucose; 2hPG, 2-hour blood glucose; FPG_25-20_, fasting plasma glucose change between 20 and 25 weeks; 1hPG-FPG, the difference between 1-hour and fasting plasms glucose levels; 1hPG-2hPG, the difference between 1-hour and 2-hour plasma glucose levels; 2hPG-FPG, the difference between 2-hour and fasting plasma glucose levels; ACU, the Areas Under Curves; FIns, fasting insulin levels; 1hIns, insulin levels after a 75 g glucose load at 1 hour; 2hIns, insulin levels after a 75 g glucose load at 2 hours; HOMA-IR, homeostatic model assessment for insulin resistance; W_25_, maternal weight at 25 weeks; W_delivery,_ maternal weight at delivery weeks; BMI_25_, body mass index at 25 weeks; BMI_delivery_, body mass index at delivery weeks; W_25-20,_ maternal weight gain between 20 and 25weeks; BMI_25-20_, body mass index gain between 20 and 25 weeks; WPW, weigh gain per week *P <0.05, **P <0.01.

The Spearman correlation heatmap analysis performed on the key differential bacteria and clinical indicators after intervention in the control and the fiber group is observed in **Figure 4**. In total, 9 species were associated with glucose metabolism levels in the control group, which was significantly more than the 3 species found in the fiber group; 4 species were associated with weight values, but none of them were seen in the fiber group. Additionally, the relative abundances of *Lactobacillaceae* and *Lactobacillus* were both positively correlated with the postprandial glucose and weight levels in the control group. Comparing the two groups, *Actinobacteriota, Actinobacteria, Bifidobacterium, Bifidobacteriales*, and *Bifidobacteriaceae* were negatively correlated with the FPG in the control group, but not significantly correlated in the fiber group. Negative correlations were identified independently between the weight values and the relative abundances of *Family XIII UCG-001and Lachnospiraceae* in the control group. Nevertheless, no correlation of significance was perceived in the fiber group.

### 3.8 Functional pathways in the gut microbiota

**Figure 5:**
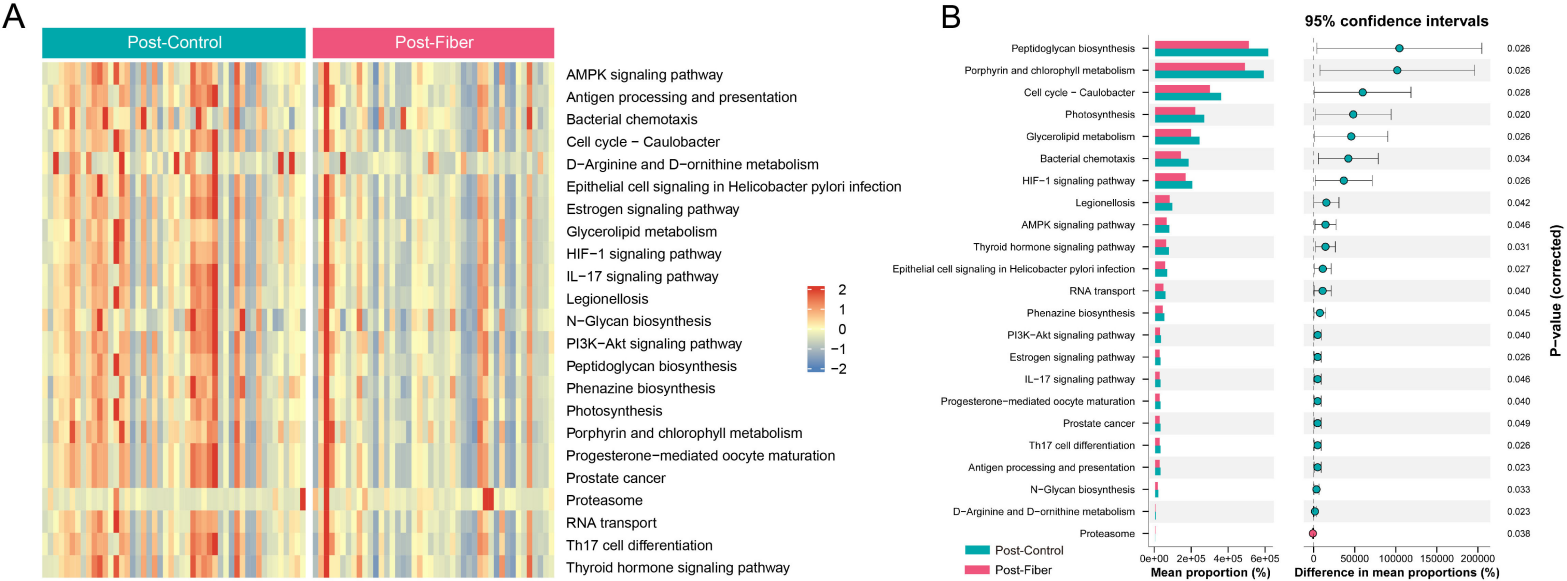
Prediction and analysis of microbial functional pathways based on the KEGG database. **(A)** The heatmap for distribution of KEGG functional pathway abundance. Red color indicates high abundance, and blue color indicates low abundance. **(B)** Comparison of predicted microbial function among groups after intervention based on KEGG level-3. KEGG, Kyoto Encyclopedia of Genes and Genomes.

Subsequently, we investigated the potential functional differences in gut bacteria responding to dietary fiber based on PICRUSt 2 analysis. We screened out a total of 23 KEGG pathways with significant differences, as shown in **Figure 5**. A t-test analysis was conducted on the abundance of annotated KEGG level-3 and showed that gut microbiota enriched in the control group compared with the fiber group were involved in 22 metabolic pathways, including peptidoglycan biosynthesis, porphyrin and chlorophyll metabolism, cell cycle, photosynthesis, glycerolipid metabolism, bacterial chemotaxis, the HIF-1 signaling pathway, legionellosis, the AMPK signaling pathway, etc. (all *P* < 0.05). In contrast, only one functional pathway within the gut microbiota in the fiber group was enriched compared to the control group, which was predominantly associated with the proteasome (*P* < 0.05) (**Figure 5B**).

### 3.9 Microbiome-enhanced prediction of GDM

**Figure 6:**
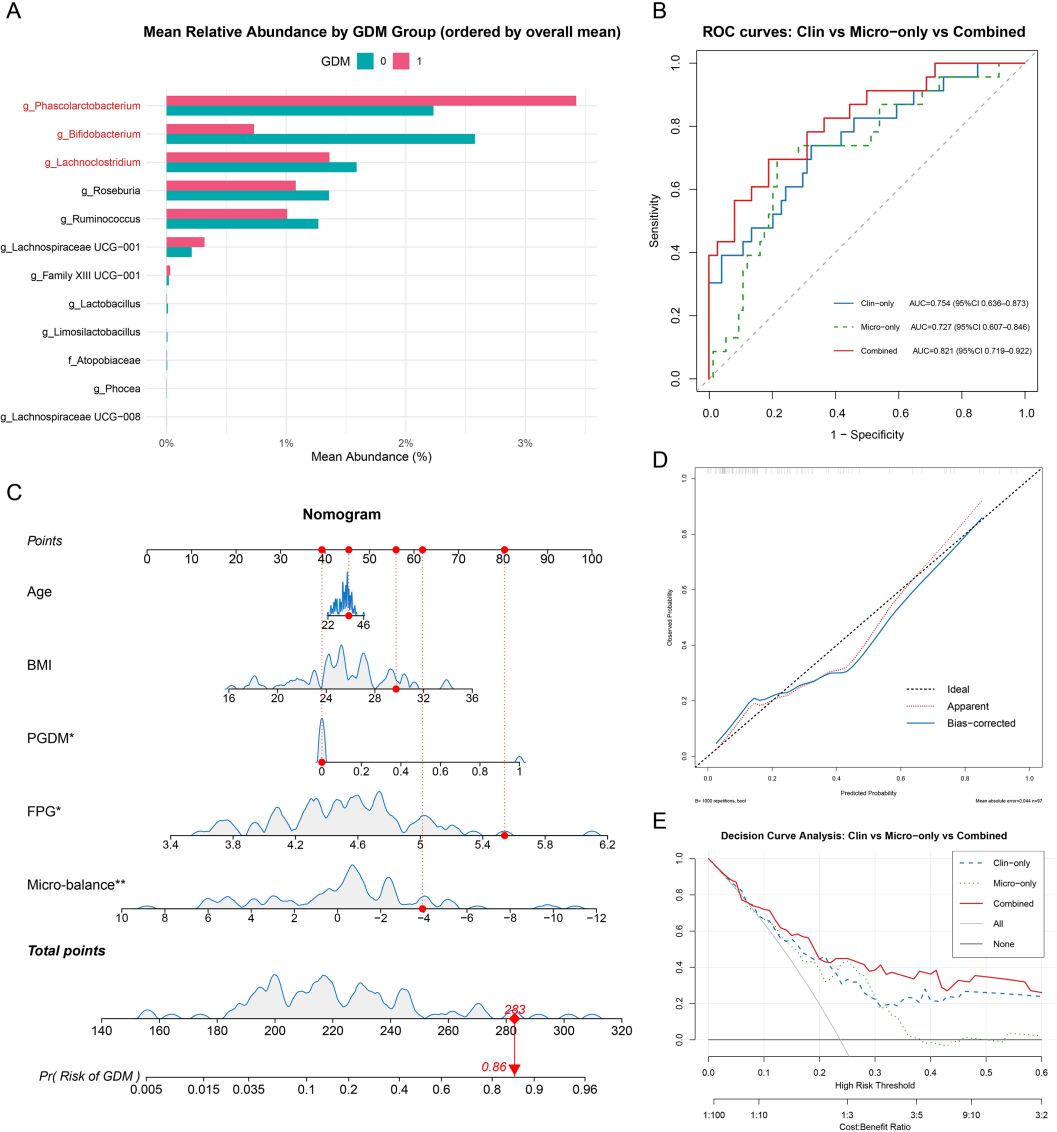
Nomogram for predicting the probability of detecting GDM patients before intervention. **(A)** Baseline mean relative abundance of selected genera by GDM status, ordered by the overall mean across all samples. **(B)** ROC curves comparing the Clin-only, Micro-only, and Combined models; the AUC (95% CI) for each model is shown in the legend. **(C)** Nomogram for the Combined model incorporating Age, BMI, PGDM, FPG, and the micro-balance feature. Kernel density rugs illustrate the distribution of each predictor. **(D)** Bootstrap-corrected calibration curve of the Combined model (apparent, optimism-corrected, and ideal lines). **(E)** Decision curve analysis (DCA) comparing net benefit for Clinical-only, Microbiome-only, and Combined strategies across threshold probabilities. PGDM, previous GDM; FPG, fasting plasma glucose *P <0.05, **P <0.01.

To assess the predictive value of fiber-responsive microbiota for GDM, we integrated a microbiome feature with key clinical indicators for early risk prediction. At baseline (**Figure 6A**), the three most abundant genera were *Phascolarctobacterium*, *Bifidobacterium*, and *Lachnoclostridium*; the first and third were relatively higher in women who developed GDM, whereas *Bifidobacterium* was more abundant in non-GDM. As one of the most abundant taxa among the differentially enriched genera we identified, *Bifidobacterium* served as the numerator of the log-ratio micro-balance used in modeling. In discrimination analyses (**Figure 6B**), the combined clinical–microbiome model performed best (AUC 0.821; 95% CI 0.719–0.922), outperforming both the clinical-only model (AUC 0.754; 95% CI 0.636–0.873) and the microbiome-only model (AUC 0.727; 95% CI 0.607–0.846).

Based on clinical and microbiome predictors of GDM risk, we developed a nomogram integrating age, BMI, prior GDM, FPG, and the micro-balance to provide individualized risk estimates (**Figure 6C**). The Combined model demonstrated good calibration (**Figure 6D**). Bootstrap-corrected metrics showed a mean absolute error (MAE) of 0.044, a mean squared error (MSE) of 0.00285, and a 90th-percentile absolute error of 0.078, indicating that 90% of predictions were within ±7.8 percentage points of observed risk. Decision-curve analysis indicated that the Combined model provided the highest net benefit across clinically relevant threshold probabilities (approximately 10-30%), exceeding the Clin-only, Micro-only, “treat-all,” and “treat-none” strategies (**Figure 6E**).

### 3.9 Adverse effects and adherence

Eight (14.8%) of 54 fiber group participants reported bloating, dizziness, diarrhea, and abdominal pain. One subject experienced mild diarrhea, one subject experienced slight dizziness, seven experienced mild bloating, and two discontinued the trial due to mild to moderate abdominal pain. Furthermore, there were no reported adverse events related to the pregnant woman’s or newborn’s health.

Of the women who completed the study, 83.3% (40/48) took 95% or more of the supplied dietary fiber powder, while 8.3% (4/48) consumed between 55% and 80%. The average consumption of the powder was 96.6% (SD 8.2%).

## 4 Discussion

In pregnant women at high risk for GDM, the effects of dietary fiber on reducing the risk of GDM and modulating gut microbiota were investigated in the present study. According to our findings, increasing the consumption of fiber supplements from 20 to 24^+6^ gestational weeks led to notable enhancements in plasma glucose levels, weight management, and a reduction in the incidence of preterm birth. Furthermore, the potential positive impact of dietary fiber on research may be dependent upon maintaining bacterial diversity and composition.

Dietary fiber, known as the “seventh nutrient,” positively affects plasma sugar levels and lipid metabolism (21). While previous studies in non-pregnant populations have shown that soluble fiber blunts postprandial glucose spikes (22, 23), our study confirms this protective effect specifically in high-risk pregnant women. Consistent with the literature, women in the fiber group exhibited significantly lower values of 1hPG, AUCs, and iAUCs compared with the control group (all *P* < 0.05). However, fiber did not significantly improve basal insulin resistance (HOMA-IR). This suggests that in our cohort of high-risk pregnancies, the primary glycemic benefit of soluble fiber lies in delaying intestinal glucose absorption and flattening postprandial glycemic excursions rather than fundamentally reversing systemic insulin resistance within a short 5-week window.

It is commonly believed that dietary fiber promotes satiety and delays gastric emptying. Mechanistically, the fermentation of dietary fiber into short-chain fatty acids (SCFAs) by gut bacteria regulates the secretion of GLP-1 and peptide YY, which decrease appetite and increase energy expenditure (24, 25). This mechanism perfectly aligns with our observation during the strict 5-week intervention window (20 to 24 + 6 weeks), where the fiber group gained significantly less weight than the control group (1.83 vs. 2.54 kg; *P* = 0.016), and the same results were shown in the BMI values (0.71 ± 0.48 vs. 1.01 ± 0.64; *P* = 0.011). Interestingly, when evaluating total gestational weight gain over the entire pregnancy, a higher percentage of women in the fiber group (52.1%) ultimately exceeded the IOM recommended limits compared to the control group (34.0%). This discrepancy can be largely attributed to the significantly extended gestational duration observed in the fiber group (39.04 vs. 38.33 weeks, *P* = 0.004), which naturally provided a longer temporal window for weight accumulation in the third trimester. Furthermore, considering that these high-risk women were already prone to excessive weight gain (many being overweight at baseline), the 5-week fiber intervention, while effectively blunting the mid-pregnancy weight spike, was not sustained long enough to offset the total weight accrued over the entire, extended pregnancy.

Epidemiological studies have demonstrated that adequate dietary fiber intake during pregnancy is essential for both maternal and fetal health, including lowering the risk of pre-eclampsia, macrosomia, and achieving appropriate gestational weight gain (26). In our study, the GDM high-risk pregnant women in the fiber group significantly extended the gestational weeks at delivery compared to women in the control group (39.04 ± 0.9 vs. 38.33 ± 1.47; *P* = 0.004), and the preterm birth (<37 weeks) incidence of women in the intervention group was also lower than the control group (0.0% vs. 12.2%; *P* = 0.040). Thus, given the efficacy of fiber in preventing preterm birth, future research and the development of preventative strategies for preterm birth are encouraged. The rate of gestational hypertension in the control group was higher than that in the fiber group but not significant (6.0% vs. 2.1%; *P* = 0.639). Furthermore, the present study did not find the benefits of fiber in lowering the risk of pre-eclampsia, excessive weight gain, and other adverse pregnancy outcomes (all *P*>0.05), which might be due to the limitations of the sample size.

Dietary fiber is well known for its ability to shape the composition and diversity of the gut microbiota. *Firmicutes* and *Bacteroidetes* represent the dominant phyla, maintaining host energy balance (27). While previous observational studies have linked a higher *Firmicutes/Bacteroidetes* (F/B) ratio and altered α-diversity to obesity and GDM onset (28, 29), our intervention yielded specific remodeling effects in this high-risk cohort. Our research found that the fiber group’s pre- and post-intervention *Bacteroides* proportion increased from 41.8% to 45.2%, while the control group’s decreased from 46.1% to 44.7%. The intervention group’s mean F/B values decreased from 1.87 to 1.46, while the control group’s increased from 1.77 to 2.16. Following the intervention, the control group had a significantly higher Chao1 index than the fiber group (*P* = 0.011). In the context of pregnancy, an abnormal surge in microbial richness (as seen in the control group) may reflect the pathological dysbiosis and metabolic stress associated with late-pregnancy insulin resistance. Fiber supplementation appeared to stabilize this microbial architecture, preventing the detrimental shifts characteristic of GDM progression. Besides *Firmicutes* and *Bacteroidetes*, *Actinobacteriota* was one of the other major phyla, Yan et al. (30) suggested that a reduction in *Actinobacteriota* may contribute to GDM. Likewise, the present study indicated that increased fiber intake significantly increased the relative abundance of *Actinobacteriota* compared with women intervention with standard prenatal care. The *Bifidobacterium* genus belongs to the *Bifidobacteriaceae* family, *Bifidobacteriales* order, *Actinobacteria* class, and *Actinobacteriota* phylum. It has a probiotic effect and is advantageous to the effect of glycemic management in pregnant women with GDM (31). Similarly, studies have shown that pregnant women with GDM have a gut microbiome imbalance compared to normal pregnant women, with reduced numbers of *Bifidobacterium* in the gut (32). In addition, Dahl C et al. investigated the impact of maternal gut microbiota on preterm birth, and discovered that the lower abundance of *Bifidobacterium* was related to spontaneous preterm delivery (33). Consequently, our findings demonstrated that obtaining a sufficient amount of fiber during pregnancy significantly increased the relative abundance of *Bifidobacterium*, and the LEfSe analysis identified the *Actinobacteriota* phylum, *Actinobacteria* class, *Bifidobacteriales* order, *Bifidobacteriaceae* family, and *Bifidobacterium* genus as the species that were differentially abundant in the fiber group as compared with the control group. Furthermore, the spearman correlation heatmap analysis indicated that *Actinobacteriot*, *Actinobacteria, Bifidobacterium*, *Bifidobacteriales*, and *Bifidobacteriaceae* abundances were all negatively correlated with the FPG values, and the fiber group exhibited notably reduced rates of preterm birth compared to the control group in terms of the maternal and fetal outcomes. Therefore, the alterations in *Bifidobacterium* may play a key role in improving glucose metabolism and reducing the occurrence of preterm deliveries.

The *Ligilactobacillus* genus and *Lactobacillaceae* family are part of the *Firmicutes* phylum, and are commonly used as probiotics. Studies have noted that an increase in the relative abundances of these two species can lead to obesity or weight gain, as they enhance nutrient absorption and energy extraction in the host (34). Additionally, the enrichment of *Lactobacillaceae* could potentially enhance insulin sensitivity and improve glucose and lipid metabolism (35). Through the analysis of the differential microbiota correlation heatmap, our findings partly align with previous reports. It was observed that *Lactobacillacea*e and *Lactobacillu* exhibited positive correlations with maternal weight gain levels; however, they were also negatively correlated with the glucose metabolism, and insulin sensitivity. This could be attributed to the study’s inherent bias—that is, the fact that all of the participants are high-risk groups for GDM. Finally, using PICRUSt 2, the metabolic function prediction of the intestinal microbiota revealed significant enrichments in glycerolipid metabolism, the HIF-1 signaling pathway, and the AMPK signaling pathway in the control group compared to those in the fiber group. Meanwhile, the control group showed significantly less enrichment in the proteasome. Interestingly, the control group displayed numerous functional pathway changes, whereas the fiber group remained relatively stable. We hypothesize that this modification reflects the natural, progressive metabolic adaptations occurring as normal pregnancy advances into the third trimester, which is typically accompanied by drastic physiological shifts such as increased insulin resistance, altered lipid metabolism, and low-grade inflammation. In contrast, dietary fiber supplementation may have exerted a stabilizing effect on the gut microenvironment, thereby blunting these dramatic functional shifts and maintaining metabolic homeostasis. Among these metabolic pathways, glycerolipid metabolism is a vital lipid metabolism pathway for lipogenesis, which is related to the regulation of fat metabolism and insulin resistance (36); the HIF-1 signaling pathway is a well-known regulator of cellular glucose and energy metabolism in pathophysiological processes; Zhu et al. found it had significant positive associations with GDM, but the actual role remains elusive (37); the pathophysiology of GDM involves the AMPK signaling system, which is also involved in controlling trophoblast mTOR activity in pregnancies complicated by fetal growth restriction and GDM involving large-for-gestational-age newborns (38). In addition, in the proteasome pathway, in response to glucose deprivation, AMPK activation suppresses the increase in 26S proteasome activity caused by high glucose. However, further investigation is required to determine the specific molecular processes of glucose and lipid metabolism under the regulation of gut bacteria (39).

Beyond analyzing individual taxa, this study represents a novel attempt to integrate microbial signatures into clinical risk stratification for GDM. While traditional risk factors (age, BMI, family history) are widely used for screening, their predictive accuracy remains suboptimal. Our analysis revealed that a specific ‘micro-balance’—defined by the log-ratio of *Bifidobacterium* to *Phascolarctobacterium* and *Lachnoclostridium*—served as a potent biomarker. The Combined Model (Clinical factors + Micro-balance) yielded an AUC of 0.821, superior to the Clinical-only model (AUC 0.754). This improvement suggests that the gut microbiome captures distinct pathophysiological information regarding host metabolic status that is not reflected by anthropometric or historical data alone. The nomogram developed herein demonstrates good calibration and clinical utility (via Decision Curve Analysis), proposing that non-invasive stool sampling in early-to-mid pregnancy could refine the identification of high-risk women who would benefit most from targeted nutritional interventions. This aligns with the growing trend of precision medicine in obstetrics, where biological markers are leveraged to tailor prophylactic strategies.

This study has several strengths. First, its randomized controlled design provides robust evidence for the effects of dietary fiber supplementation. Second, follow-up spanned most of pregnancy, enabling assessment of sustained impacts on glycemic control, gestational weight gain, and maternal–neonatal outcomes. Third, we observed post-intervention increases in taxa associated with glucose homeostasis and weight regulation, providing a biologically plausible basis for future probiotic strategies in GDM prevention and maternal-fetal health. Nonetheless, several disadvantages should be considered. First, the study’s statistical power is hampered by its single center and small sample size (N = 98). This caution should be applied to any further subgroup studies, and the generalizability of these findings requires further evaluation. Second, 16S rRNA sequencing provides a good image of the microbiota right down to the genus level but not usually the species level. More metagenomic shotgun sequencing is needed to confirm species and strain taxonomy. Third, precise food consumption data and microbiota-derived metabolites (e.g., short-chain fatty acids) were not measured, limiting mechanistic interpretation of how nutrition impacts the gut microbiome and its metabolic outputs. To confirm these findings, multicenter cohorts with bigger sample sizes and integrated metagenomics/metabolomics should be used.

## 5 Conclusions

In general, dietary fiber supplementation in high-risk pregnancies showed potential in supporting 1hPG homeostasis, while potentially mitigating excessive weight gain and lowering preterm birth risk. Our preliminary findings suggest these benefits may be associated with the modulation of specific gut commensals, particularly *Bifidobacterium.* Furthermore, integrating microbiome signatures with clinical metrics shows promise for enhancing GDM risk prediction. Future validation in larger, multi-center cohorts is necessary to confirm these findings and fully realize the potential of precision nutrition in prenatal care.

## Data Availability

The datasets presented in this study can be found in online repositories. The names of the repository/repositories and accession number(s) can be found below: https://www.ncbi.nlm.nih.gov/, PRJNA1408403.
